# The Year in Cardiology 2015: Heart Failure

**DOI:** 10.1093/eurheartj/ehv720

**Published:** 2016-08

**Authors:** Michel Komajda, Frank Ruschitzka

**Affiliations:** 1 Department of Cardiology, Pitié-Salpêtrière Hospital, University Pierre and Marie Curie and IHU ICAN, Paris, France; 2 Department of Cardiology, University Heart Centre, University Zurich, Zurich, Switzerland

**Keywords:** Heart Failure/complications, Sleep Apnea, Obstructive/therapy, Sleep Apnea, Central/therapy, Positive-Pressure Respiration, Cardiovascular Diseases/mortality

## Preamble

A number of studies conducted both in heart failure with reduced and with preserved ejection fraction were presented and published in 2015. Most of them were neutral and did not demonstrate any benefit on outcomes of the drugs/procedures tested. Nevertheless, they bring important new information on the search for new drugs or procedures in the management of heart failure.

### Adaptive servo-ventilation in heart failure and central sleep apnoea: is it harmful?

Sleep-disordered breathing is common in patients with heart failure and reduced ejection fraction. Two different types of abnormality have been described: obstructive sleep apnoea and central sleep apnoea. The prevalence of central sleep apnoea, which may manifest as Cheynes-Stokes respiration, increases with the severity of heart failure and this condition is associated with poor outcomes.

The purpose of SERVE-HF was to assess the effects of adaptive servo-ventilation (ASV) that delivers servo-controlled inspiratory pressure support on top of expiratory positive airway pressure in patients with moderate to severe heart failure and an ejection of < 45% who had predominantly central sleep apnoea.^1^ In this trial, 1325 patients were enrolled and randomized to ASV (666) or to control therapy (659). Patients were predominantly in New York Heart Association Class III and were well treated by recommended therapies. The incidence of the primary endpoint made of the composite of death of any cause, lifesaving cardiovascular intervention, or unplanned hospitalization for heart failure did not differ significantly between the two groups (HR = 1.13; 95% CI, 0.97-1.31; p = 0.10). The surprise was the observation of a significant increase of all-cause mortality (HR = 1.28; 95% CI, 1.06-1.55; p = 0.01) and of cardiovascular mortality (HR = 1.34; 95% CI, 1.09-1.65; p = 0.006) in the ASV group. The findings of SERVE-HF contrast with evidence from earlier smaller studies that suggested an improvement in left ventricular function, quality of life, and mortality.

One potential explanation for the increase in cardiovascular mortality is that central sleep apnoea may be a compensatory mechanism, and therefore reducing this adaptive respiratory pattern by ASV may be detrimental. The other explanation put forward by Cowie et al.^1^ is that the application of positive airway pressure may impair cardiac function, in particular, in patients with low pulmonary capillary wedge pressure. The timing of death and whether the fatal events occurred while patients were under ASV will be therefore important to determine the potential mechanism of harm.

One important implication of the negative results of SERVE-HF is that this procedure should not be recommended anymore for patients with heart failure and reduced ejection fraction and central sleep apnoea and stopped in those patients currently treated by this procedure. This, however, does not apply to obstructive sleep apnoea.

Whether other techniques diminishing Cheynes-Stokes respiration such as phrenic nerve stimulation are beneficial or harmful remains an open question until the results of the ongoing trial testing phrenic nerve stimulation are available.

### Glucose-lowering agents and risk of heart failure: new and reassuring results

Dipeptidyl peptidase 4 inhibitors (DPP4 inhibitors) have been used for several years in the management of type 2 diabetes mellitus. In 2013, the publication of SAVOR-TIMI 53 raised concern on the safety of this class regarding the occurrence of heart failure events.^2^ This large outcome trial including patients with diabetes mellitus and a previous cardiovascular event or at high cardiovascular risk showed that the overall cardiovascular safety of saxagliptin was good, except a 27% increase in the risk of the first event worsening heart failure hospitalization. There was no biological plausible explanation for this observation. Nevertheless, this raised concern on potential harm all the more as another trial EXAMINE conducted in patients with diabetes mellitus and presenting with an acute coronary syndrome suggested a non-significant signal for increased risk of heart failure with another DPP4 inhibitor, alogliptin (Table 1).^3^

**Table 1:** Heart failure events in recent trials with glucose-lowering drugs

**Study**	**Drug**	**No. of Patients**	**Follow-up (years)**	**Heart failure (HR) hospitalization**	**P-value**
SAVOR	Saxagliptin	16 492	2.1	1.27 (95% CI, 1.07-1.51)	0.007
EXAMINE	Alogliptin	5380	1.5	1.07 (95% CI, 0.79-1.46)	0.66
TECOS	Sitagliptin	14 671	3.0	1.00 (95% CI, 0.83-1.20)	0.98
EMPA-REG	Empagliflozin	7020	3.1	0.65 (95% CI, 0.50-0.85)	0.002

The publication of TECOS, another mega trial including 14 671 patients was therefore long awaited.^4^ Patients included had type 2 diabetes mellitus, were 50 years of age or more, and had an established cardiovascular disease and a baseline HbA1C of 6.5-8%. They were randomized to either the DPP4 inhibitor sitagliptin or to control treatment.

After 3 years of follow-up, no difference was observed in the occurrence of the composite endpoint of cardiovascular mortality, non-fatal myocardial infarction, non-fatal stroke, or hospitalization for unstable angina (HR = 0.98; 95% CI, 0.88-1.09; p < 0.001 for non-inferiority). Importantly, the incidence of heart failure was similar in the two arms with a hazard ratio of 1.00 (95% CI, 0.83-1.20; p = 0.98). The explanation for the differential effect of sitagliptin and of saxagliptin on heart failure events remain uncertain: differences in populations enrolled in the two trials are unlikely to play a role since the clinical profile of the patients were rather similar. Differences in affinity of the two inhibitors to the various substrates of DPP4 are a potential explanation. Finally, the play of chance cannot be excluded in this very large trial.

Whatever the underlying explanation, the results of this large outcome trial in type 2 diabetes mellitus rule out a class effect of DPP4 inhibitors on heart failure events and are therefore reassuring regarding the safety of sitagliptin in patients with pre-existing heart failure or at high risk of heart failure.

Another trial, EMPA-REG OUTCOME, tested two doses of an inhibitor of sodium-glucose co-transporter 2, empagliflozin vs. placebo in 7020 patients with type 2 diabetes at high cardiovascular risk.^5^ After a median observation time of 3.1 years, the primary outcome made of death from cardiovascular causes, non-fatal myocardial infarction, or non-fatal stroke was significantly reduced by 14% in the pooled empagliflozin group. Interestingly, hospitalizations for heart failure and the composite of hospitalization for heart failure or death from cardiovascular causes, two secondary endpoints, were also significantly reduced by 35% (p = 0.002) and 34% (p < 0.01), respectively, suggesting that this new anti-diabetic agent added to standard therapy is not only safe but also beneficial for the prevention of heart failure hospitalizations in type 2 diabetes mellitus.

### Management of heart failure with preserved ejection fraction remains a clinical dilemma

**Figure 1:**
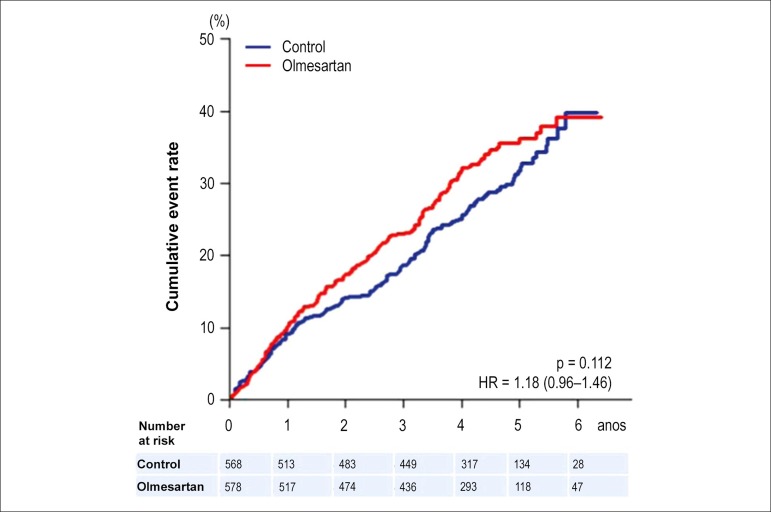
Kaplan Meier curve for the primary composite endpoint (all cause death, non fatal myocardial infarction, non fatal stroke and hospitalization for worsening heart failure) in the overall SUPPORT population.^6^

The medical management of heart failure with preserved ejection fraction (HFpEF) remains challenging, and no drug has demonstrated a clear benefit on morbidity and mortality in this population (Figure 1).

This Figure has been reprinted by permission of Oxford University Press on behalf of the European Society of Cardiology.

The SUPPORT trial examined whether an additive treatment with an angiotensin receptor blocker, olmesartan, reduces the mortality and morbidity in hypertensive patients with chronic heart failure treated with angiotensin-converting enzyme (ACE) inhibitors, beta blockers, or both. In this prospective randomized open-label study, 1147 patients were enrolled.^6^ Mean ejection fraction was 54%. During a median follow-up of 4.4 years, there was no statistical difference in the occurrence of the primary outcome made of all-cause death, non-fatal myocardial infarction, non-fatal stroke, and hospitalization for worsening heart failure between the two groups (HR = 1.18; 95% CI, 0.96-1.46; p = 0.11), whereas a significant increase in worsening renal function was observed. The addition of olmesartan to patients treated by the combination of ACE inhibitors and beta blockers was, however, associated with a significant increase in the occurrence of the primary endpoint (HR = 1.47; 95% CI, 1.11-1.95; p = 0.006) all-cause death and renal dysfunction. These findings lead to the conclusion that combination therapy of ACE inhibitors, angiotensin receptor antagonists, and beta blockers is not recommended in HFpEF since it is associated with increased cardiovascular risk and increased risk of renal dysfunction.

In 2013, the RELAX trial conducted in 216 elderly patients with HFpEF showed the absence of effect of the phosphodiesterase type 5 sildenafil on maximal exercise capacity, 6 min walking distance, clinical status, quality of life, left ventricular remodelling or diastolic function after 24 weeks of follow-up.^7^ These results were in contrast with a previous single centre study that showed benefit on invasively measured haemodynamics, echocardiographic variables, and quality of life in patients with pulmonary hypertension related to HFpEF.^8^

Another just recently published single centre study by Hoendermis et al.^9^ published in the European Heart Journal, however, casts further doubt on the use of sildenafil in HFpEF patients with associated pulmonary hypertension. Fifty-two patients with HFpEF and predominantly isolated post-capillary pulmonary hypertension were randomized to sildenafil or placebo. After 24 weeks, sildenafil did not reduce pulmonary artery pressures and did not improve other invasive haemodynamic or clinical parameters, thus confirming the findings of the aforementioned RELAX study that HFpEF patients with associated pulmonary hypertension do not benefit from treatment with this drug.

The current paradigm of HFpEF is that an abnormal nitric oxide bioavailability results in decreased cyclic guanylate monophosphate (cGMP) in the myocytes. One potential explanation of the lack of benefit from sildenafil is therefore that the defect is more a decrease in the production of cGMP than a problem of increased degradation that is inhibited by PDE5 inhibitors such as sildenafil. It will therefore be interesting to see the results of studies using a soluble guanylate cyclase (sGC) stimulator, such as riociguat, which is currently under evaluation. The results of the SOCRATES-REDUCED study, however, highlight the challenges in moving the concept of modulating sGC and thereby addressing the relative cGMP deficit forward.^10^ In SOCRATES-REDUCED, a phase 2 dose-finding study in patients with heart failure with reduced ejection fraction and worsening chronic HF, the oral sGC stimulator vericiguat did not meet its primary endpoint of reducing N-terminal pro-B-type natriuretic peptide (NT-proBNP) at 12 weeks when all doses were combined, but was well tolerated. While subgroup analysis did suggest efficacy and safety in its 10 mg subgroup, further studies are needed to determine the potential role of this class of drugs for patients with worsening chronic HF.

The current paradigm that increasing nitric oxide bioavailability may provide meaningful net clinical benefit was further questioned by the just recently published results of the multicentre, double-blind, placebo-controlled Nitrate's Effect on Activity, Tolerance in Heart Failure with Preserved Ejection Fraction (NEAT-HFpEF) trial.^11^

In this National Heart, Lung, and Blood Institute-sponsored trial, 110 patients with heart failure and preserved ejection fraction were randomly assigned to a 6-week dose-escalation regimen of isosorbide mononitrate (from 30 to 60 mg to 120 mg once daily) or placebo, with subsequent crossover to the other group for 6 weeks. Intriguingly, at every tested nitrate dose patients with HFpEF had lower levels of activity and did not have better quality of life or submaximal exercise capacity than patients taking placebo. Of note, no interaction between the subgroups, including by age, sex, heart failure aetiology, natriuretic peptide levels, or blood pressure, was observed.

It is intriguing to speculate whether other Nitric Oxyde donors than isosorbide mononitrate, such as inorganic nitrite or nitrate (which have been shown to increase nitric oxide bioavailability during exercise), might have yielded more beneficial results under the conditions of the study. This notwithstanding, the somewhat counterintuitive findings of NEAT-HFpEF once again highlight the distinct pathophysiologic differences between HFpEF vs. heart failure with reduced ejection fraction (HFrEF). Indeed, since long-acting nitrates improve symptoms in HFrEF, the results of NEAT-HFpEF therefore suggest that the potential haemodynamic benefits of nitrates are less likely to come into play under the conditions of increased ventricular systolic and vascular stiffness, autonomic dysfunction, chronotropic incompetence, and altered baroreflex sensitivity as they are common in in patients with HFpEF.

### Angioedema and angiotensin-converting enzyme inhibitors

Angioedema is a rare but potentially life-threatening side effects of ACE inhibitors and there is no approved treatment. It is generally related to the inhibition of the degradation of bradykinin, therefore increasing the activity of this peptide. A phase 2 study compared the effects of subcutaneous icatibant, a selective bradykinin B2 receptor antagonist to intravenous prednisolone plus an antihistaminic agent, clemastine, in 27 patients who had ACE-induced angioedema of the upper aerodigestive tract.^12^ Icatibant induced a complete resolution of symptoms in 8 h on average compared with 27 h with standard therapy.

These results suggest that the use of a bradykinin receptor antagonist allows complete resolution of ACE inhibitors induced angioedema faster than with the standard therapy.

### Alcohol consumption and risk of heart failure

**Figure 2:**
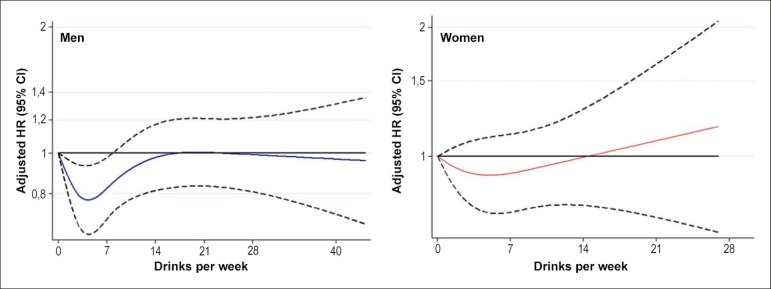
Relative risk of incident heart failure as a function of alcohol intake at baseline by sex. The 95% confidence intervals are indicated by the dash lines. Models are adjusted for age, diabetes, hypertension, Coronary artery disease, body mass index, total cholesterol physical activity, education level, smoking status and incident myocardial infarction as time-varying covariate.^13^

Heavy alcohol consumption is associated with cardiac dysfunction and eventual alcoholic cardiomyopathy (Figure 2). However, the relationship between moderate alcohol intake and risk of heart failure is controversial. Self-reported alcohol consumption was assessed in 14 629 participants of the Atherosclerosis Risk in Communities (ARIC) study without prevalent heart failure at baseline.^13^ During an average follow-up of 24 years, incident heart failure occurred in 1271 men and 1237 women. Men consuming up to 7 drinks a week (one drink = 14 g of alcohol) had a reduced risk of heart failure relative to abstainers (HR = 0.80; 95% CI, 0.68-0.94; p = 0.006). This 'protective' effect was less robust in women (HR = 0.84; 95% CI, 0.71-1.00; p = 0.05). In the heavy drinking categories, the risk of heart failure was not different from abstainers either in women or in men. These results suggest therefore that modest alcohol consumption may be associated with a lower risk of heart failure.

This Figure has been reprinted by permission of Oxford University Press on behalf of the European Society of Cardiology.

### Gene therapy in chronic heart failure: disappointment

Cardiac regeneration using gene transfer in the myocardium is a novel approach to the treatment of heart failure. Abnormal calcium cycling in the cardiomyocyctes is a hall mark of moderate to severe heart failure, and one key element is deficient expression and activity of sarcoplasmic reticulum Ca2+ ATPase type 2a (SERCA2a), the molecule that pumps calcium from the cytosol to the intracellular stores, i.e. the sarcoplasmic reticulum. Preclinical studies have shown that the increased expression of SERCA2a in cardiomyocytes normalizes calcium cycling and that SERCA2a gene transfer in large animal models can reverse cardiac dysfunction. CUPID 2 enrolled 250 patients with severe heart failure who received intracoronary either the transgene (123) or placebo (127).^14^ The primary endpoint was time to recurrent heart failure-related hospitalizations and ambulatory worsening heart failure in presence of terminal events, including all-cause death or transplant. There was no difference between the active and the conventional arms for the primary endpoint (HR = 0.93; 95% CI, 0.53-1.65; p = 0.81) or for any of the secondary endpoints. No safety issue was raised during the trial. These disappointing results have no clear explanation and are in particular in contradiction with a previous smaller trial (CUPID), which suggested that intracoronary injection of SERCA2a transgene was associated with a dose-dependent beneficial effect on ventricular function, patient well-being, and biomarkers at 6 and 12 months and that outcomes were improved at 3 years in the patients treated with the high dose. Potential explanations for failure include dose of the transgene, mode of injection, durability of the effect, type of vector (here an adenovirus) and promoter (cytomegalovirus), or the target. It is hoped that these negative results will not freeze research in this area and that different approaches including more cardio specific promoters, mode of injection, or vectors will be tested to better assess the potential role of gene transfer for cardiac regeneration.

Treatment of Chagas' cardiomyopathy by benznidazole

Chagas'disease is a common parasitic disease in Latin America and is responsible for the most common form of non-ischaemic cardiomyopathy in this area. Chagas'cardiomyopathy develops in 25% of patients infected by Trypanosoma cruzi 20-30 years after the acute infection. The role of trypanocidal therapy at the stage of Chagas'cardiomyopathy is unproven. The Benznidazole Evaluation for Interrupting Trypanosomiasis (BENEFIT) trial evaluated the effects on outcomes of oral benznidazole, a trypanocidal agent vs. placebo in 2854 patients who had evidence of Chagas'cardiomyopathy.^15^ The drug was administered for 40-80 days and patients were followed for a mean of 5.4 years. The primary outcome was time to death, resuscitated ventricular tachycardia, insertion of a pacemaker or implantable cardioverter-defibrillator, cardiac transplantation, new heart failure, stroke, or other thromboembolic event. Although trypanocidal therapy with benznidazole significantly reduced serum parasite detection by polymerase chain reaction, there was no significant effect on the primary outcome (HR = 0.93; 95% CI, 0.81-1.07; p = 0.31). Potential explanations for these negative results include genetic variations of T. cruzi, insufficient period of observation, and late treatment at a stage of advanced cardiac disease.
